# Photochemical activity in developing pea (Pisum sativum L.) cotyledons depends on the light transmittance of covering tissues and the spectral composition of light

**DOI:** 10.18699/VJGB-23-113

**Published:** 2023-12

**Authors:** G.N. Smolikova, N.V. Stepanova, A.M. Kamionskaya, S.S. Medvedev

**Affiliations:** Saint Petersburg State University, St. Petersburg, Russia Federal Research Centre “Fundamentals of Biotechnology” of the Russian Academy of Sciences, Moscow, Russia; Saint Petersburg State University, St. Petersburg, Russia Federal Research Centre “Fundamentals of Biotechnology” of the Russian Academy of Sciences, Moscow, Russia; Federal Research Centre “Fundamentals of Biotechnology” of the Russian Academy of Sciences, Moscow, Russia; Saint Petersburg State University, St. Petersburg, Russia

**Keywords:** Pisum sativum L., seed maturation, light transmittance of tissues, illumination intensity, photochemically active radiation, photochemical activity, PAM fluorometry, Pisum sativum L., созревание семян, светопропускание тканей, интенсивность освещения, фотохимически активная радиация, фотохимическая активность, РАМ-флуориметрия

## Abstract

Many crops require not only leaf photosynthesis for their seed development but also the photochemical reactions that occur in the seeds. The purpose of this work was a comparative analysis of light transmittance and photochemical activity in the leaves of Pisum sativum L. and its pericarp, seed coat, and cotyledons at the early, middle, and late maturation stages. The spectral composition of light was measured using a spectroradiometer in the range of 390–760 nm. We assessed the light transmittance of plant tissues by placing the plant tissue between the light source and the spectroradiometer’s sensor. PAM fluorometry was used to quantify the photochemical activity in plant tissues: this technique is handy for evaluating the efficiency of converting light energy into chemical energy through the analysis of the kinetics of chlorophyll fluorescence excitation and quenching. On average, a photochemically active green leaf of pea transmitted 15 % of solar radiation in the 390–760 nm, blue light was delayed entirely, and the transmitted red light never exceeded 5 %. Photochemically active radiation passing through the pericarp and coat and reaching the cotyledons at the early and middle seed maturation stages manifested a high proportion of green and far-red light; there was no blue light, and the percentage of red light was about 2 %. However, the cotyledons were photochemically active regardless of low irradiance and spectral ranges untypical of leaf photosynthesis. At the early and middle maturation stages, the maximum quantum yield of photosystem II (Fv/Fm) averaged 0.5 at the periphery of cotyledons and 0.3 at their center. Since the intensity of embryonic photochemical reactions significantly affects the efficiency of reserve nutrient accumulation, this parameter is a promising marker in pea breeding for seeds with improved nutritional qualities.

## Abstract (ru)

У многих сельскохозяйственных растений для формирования семян необходимы не только фотосинтез листьев, но также фотохимические реакции, происходящие в семенах. Цель нашей работы заключалась в сравнительном анализе светопропускания и фотохимической активности листьев, перикарпия, кожуры и семядолей Pisum sativum L. на ранней, средней и поздней стадиях созревания семян. Спектральный состав света измеряли при помощи спектрорадиометра в области 390–760 нм. Светопропускание растительных тканей оценивали, разместив растительную ткань между источником освещения и датчиком спектрорадиометра. Фотохимическую активность растительных тканей определяли методом РАМ-флуориметрии, позволяющим оценить эффективность преобразования световой энергии в химическую путем анализа кинетики возбуждения и гашения флуоресценции хлорофиллов. Фотохимически активный зеленый лист гороха пропускал в среднем 15 % солнечной радиации в диапазоне 390–760 нм; при этом синий свет задерживался полностью, а количество проходящего красного света составляло не более 5 %. Фотохимически активная радиация, проходящая сквозь перикарпий и кожуру и достигающая семядолей на ранней и средней стадиях созревания семян, характеризовалась высокой долей зеленого и дальнего красного света, при этом синий свет отсутствовал, а количество красного света составляло около 2 %. Однако, несмотря на низкую энергетическую освещенность и спектральные диапазоны, не характерные для фотосинтеза листа, семядоли были фотохимически активными. На ранней и средней стадиях созревания максимальный квантовый выход фотосистемы II (Fv/Fm) в среднем составлял 0.5 на периферии семядолей и 0.3 в центре семядолей. Поскольку интенсивность эмбриональных фотохимических реакций в значительной степени влияет на эффективность накопления запасных питательных веществ, этот параметр является перспективным маркером для селекции семян гороха с улучшенными пищевыми качествами.

## Introduction

Seed-based products represent nearly three-quarters of human food, making high-quality seed production the foundation of food security (Mattana et al., 2022). An essential factor in plant seed productivity is photosynthesis, which occurs in leaves and provides developing seeds with the necessary assimilates (Simkin et al., 2019a, 2010; Walter, Kromdijk, 2021). Therefore, most studies aimed at developing approaches that could increase crop productivity have focused on analyzing leaf photosynthetic processes. Meanwhile, other plant organs (petioles, stems, inner bark, and fruits) can also synthesize chlorophylls and develop actively functioning chloroplasts, where the so-called non-foliar photosynthesis occurs (Aschan, Pfanz, 2003; Tikhonov et al., 2017; Hu L. et al., 2019; Henry et al., 2020; Simkin et al., 2020; Yanykin et al., 2020).

The presence of green pigments in the embryos, as well as in the pericarp and seed coat of angiosperms, has been known since the middle of the 19th century (Hofmeister, 1859; Flahault, 1879; Monteverde, Lyubimenko, 1909). According to the analysis of the pigments in the maturing seeds of rape, they contained chlorophyll a, chlorophyll b, pheophytin a, pheophytin b, and, in minor amounts, pheophorbide a, methyl pheophorbide a, and pyropheophorbide (Ward et al., 1994). At the same time, the total chlorophyll content per unit of wet weight and the ratio of chlorophylls a/b were lower in green embryos than in leaves (Bulda et al., 2008; Smolikova et al., 2011, 2018, 2020). Comparison of the chlorophylls and carotenoids content in the leaves of shade-adapted plants and in the green embryos of developing oilseeds revealed it to be approximately equal (Ruuska et al., 2004).

Non-foliar green tissues of C3 plants can reassimilate CO2 released during respiration, providing up to 15–48 % of the total carbon dioxide assimilated during photosynthesis (Hu L. et al., 2019). However, the contribution of these tissues to the total amount of assimilates synthesized in the light is often ignored. Non-foliar photosynthesis can also occur in the developing seeds of many plant species (Borisjuk et al., 2003; Allorent et al., 2015; Smolikova, Medvedev, 2016; Smolikova et al., 2017, 2018, 2020; Brazel, Ó’Maoiléidigh, 2019; Hu L. et al., 2019; Grulichova et al., 2022; Shackira et al., 2022).

Embryologists from the Komarov Botanical Institute of the Russian Academy of Sciences (St. Petersburg) were the first in the world to study the genesis and structure of plastids in the embryos of more than 1,000 plant species (Yakovlev, Zhukova, 1973, 1980). They identified 428 plant species, the embryos of which contained chlorophylls and plastids with well-developed thylakoid membranes. These species became known as chloroembryophytes. Later, it has been shown that the function of the photosynthetic apparatus in the developing seeds is directed to the synthesis of storage compounds (mainly fatty acids) rather than the monosaccharides as in leaves (Neuhaus, Emes, 2000; Ruuska et al., 2004; Weber et al., 2005; Allen et al., 2009; Hu Y. et al., 2018).

Expression of nuclear genes responsible for the process of photosynthesis was observed in Arabidopsis and rapeseed embryos starting from the globular stage of embryogenesis (Spencer et al., 2007; Le et al., 2010; Belmonte et al., 2013; Kremnev, Strand, 2014). The priority function of seed chloroplasts is the rapid synthesis of NADPH and ATP, which are used to convert sucrose supplied from the mother plant into acetyl-CoA and fatty acids and further into triglycerides (Ruuska et al., 2004; Allen et al., 2009; Puthur et al., 2013; Wu et al., 2014; Allorent et al., 2015; Shackira et al., 2022). It means that reserve nutrient accumulation in seeds depends on the efficiency of embryo photochemical reactions. For example, rape (Brassica napus L.) pods shielded from light during their development had significantly decreased seed weight and proteins and fatty acids content (Wang et al., 2023).

Seed embryos are typically covered with seed and pod coats, hindering the exchange of carbon dioxide and oxygen and shielding from sunlight. A crucial aspect of photo-dependent synthetic reactions in seed embryos involves using sucrose supplied from the mother plant and CO2 released through respiration, rather than atmospheric CO2, as a carbon source (Ruuska et al., 2004). At the same time, the O2 released during photooxidation of water prevents hypoxia and supports mitochondrial respiration in developing seeds (Borisjuk et al., 2003; Weber et al., 2005; Borisjuk, Rolletschek, 2009; Tschiersch et al., 2011; Shackira et al., 2022). Recently, it has been shown in soybean (Glycine max) plants that non-foliar photosynthesis occurring in the pericarps and coats contributes up to 9 % of the total daily carbon assimilation and can compensate for up to 81 % of the carbon loss by respiration of these tissues (Cho et al., 2023). Nevertheless, in-depth studies are needed to investigate the mechanisms of photodependent synthetic reactions related to the accumulation of reserve nutrients.

Therefore, it remains unclear how developing seeds receive sufficient light to generate the energy for photochemical reactions. No detailed research has been conducted to determine the spectral characteristics of the light inside the seed embryos.

This study aimed to conduct a comparative analysis of light transmittance and photochemical activity between the leaves and tissues of developing pea seeds (pericarps, coats, cotyledons).

## Materials and methods

**Fig. 1.**
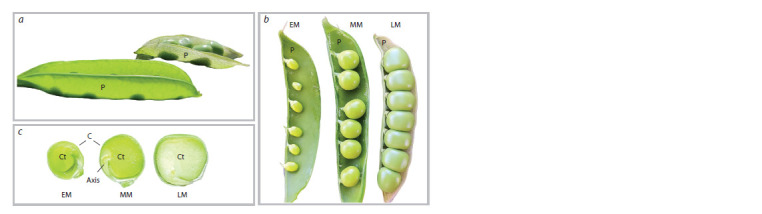
The images of pea pods and seeds at the early, middle, and late maturation stages (EM, MM, and LM, respectively). а – the photo demonstrates high light transmittance of the pericarp; b – pods with seeds; c – seeds in longitudinal section; P – pericarp; C – coat; Ct – cotyledon; Axis – embryonic axis including the root, hypocotyl, epicotyl, and plumule.

Common pea (Pisum sativum L.) plants of the vegetable cv. Prima were used as the material in this study. This cultivar was approved for cultivation in the Central and North Caucasus and was added to the State Register (National List) in 2015. Seeds are wrinkled, large-sized, with green cotyledons (Besedin, 2015). Plants were grown in outdoor plots at St. Petersburg State University during the summer of 2022 under natural lighting conditions. We examined seeds at the early, middle, and late maturation stages, as shown in Fig. 1. The early maturation stage is marked by the end of embryo development and the start of reserve nutrient accumulation in the cotyledons (Smolikova et al., 2018, 2020). At the middle maturation stage, reserve nutrients are synthesized actively, causing the cotyledons to expand and fill the inner space of the seed. Finally, the seeds lose their moisture at late maturation, develop desiccation tolerance, and enter dormancy.

The spectral composition of light was measured using the spectroradiometer TKA “Spectr” (St. Petersburg, Russia). The device detects light spectral characteristics in the spectrum’s visible range from 390 to 760 nm. The recorded irradiance spectral density was expressed in energy units per m2 (mW/m2).

We evaluated the light transmittance of plant tissues by placing the plant tissue between the light source and the sensor of the spectroradiometer. Natural solar radiation served as the source of light

The photochemical activity of plant tissues was quantified by pulse amplitude modulation (PAM)-based fluorometry using a Walz MINI-PAM-II/B (Heinz Walz GmbH, Germany) according to the manufacturer’s protocol (MINI-PAM-II: Manual for Standalone Use, 2018). The device is equipped with measuring and actinic light sources with an emission maximum of 470 nm and fluorescence detection at wavelengths > 630 nm. The measuring and active light intensities were 0.05 and 190 μM photons/(m2 · s), respectively. The saturating pulse intensity was 5000 μM photons/(m2 · s) with a duration of 0.6 s. Plant tissues (leaves, pericarps, coats, and cotyledons) were isolated from the mother plant, placed on moist filter paper (to prevent drying), and kept in light-proof boxes for 20 min for dark adaptation. Leaf clip 2030-B was used to hold the tissues. The following fluorescence ratio parameters were evaluated:

Fv/Fm, i. e., the maximum photochemical quantum yield of photosystem II (PSII) when all electron carriers in the electron transport chain (ETC) of chloroplasts are oxidized. It is detected immediately after dark adaptation of the tissue. Fv/Fm is calculated as the ratio of the light quantum used for the charge separation to the total amount absorbed by lightharvesting complexes (LHC):

**Formula. 1.**
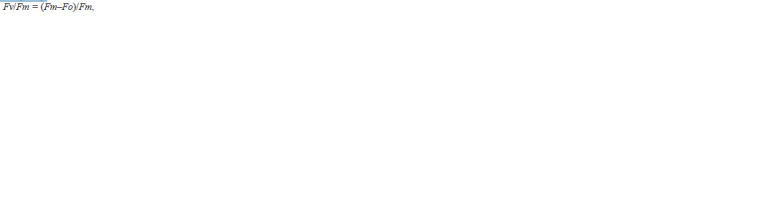
Formula. 1.

where Fо is the minimum level of fluorescence under measuring light that does not excite the transfer of electrons from donors to acceptors; Fm is the maximum level of fluorescence elicited by a saturation pulse that saturates all reaction centers (RC) of PS with electrons; Fv is the variable fluorescence, calculated by subtracting Fo from Fm.

Y(II ), i. e., the effective quantum yield of photochemical quenching, measured in the light-adapted samples:

**Formula. 2:**
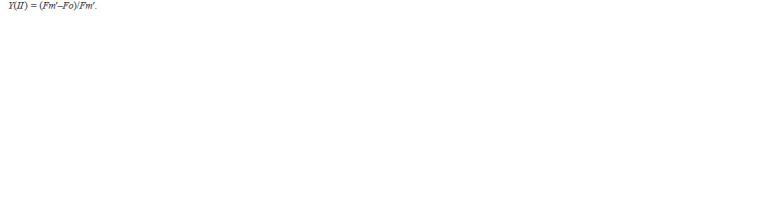
Formula. 2.

NPQ, i. e., the non-photochemical quenching of fluorescence. It is calculated using the Stern–Volmer equation, according to which fluorescence quenching is proportional to the number of quenching centers in the LHC:

**Formula. 3:**
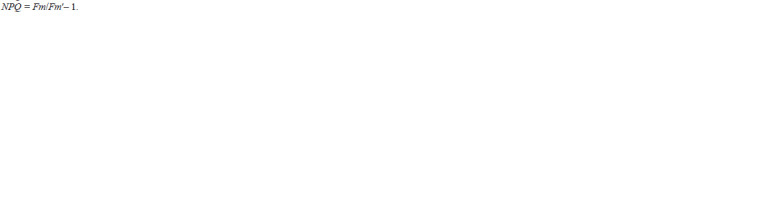
Formula. 3.

Statistical data processing and software. Three biological replicates were performed for each measurement. Quantitative chlorophyll fluorescence parameters and corresponding design ratios were obtained using the WinControl-3 program (Heinz Walz GmbH, Germany). Statistical processing was done in the Microsoft Excel 2023 software using a standard data analysis package. The graphs and tables present arithmetic means and standard deviations. All data were expressed as an arithmetic mean ± standard deviation and processed using Excel for Microsoft 365 with embedded statistical data analysis tools. A two-way analysis of variance (ANOVA) with replications was performed. Differences were considered statistically significant at a confidence level of p ≤ 0.05

## Results

We studied the dynamics of light transmission in the pericarp, seed coat, and cotyledon tissues during the seed development of pea plants. The images of pods, seeds, and embryos are shown in Fig. 1.

Light transmittance was assessed by placing the plant tissue between the sunlight and the spectroradiometer’s sensor. Solar radiation served as a control reference, taken as 100 %. We compared photosynthetically active green leaves, senescent yellow leaves, the pericarps, coats, and the summed combination of the pericarps and coats. The spectrum of solar radiation reaching the pod tissue is shown in Supplementary Material (a)1. Photosynthetically active green leaves of pea plants completely blocked blue and red light in the ranges corresponding to the chlorophyll and carotenoid absorption maxima, transmitted part of green and yellow light, and fully transmitted far-red light (see Supplementary Material, b). With leaf senescence, chlorophylls degraded, and the amount of transmitted blue and red light increased (see Supplementary Material, c).


Supplementary Materials are available in the online version of the paper: https://vavilov.elpub.ru/jour/manager/files/Suppl_Smolikova_Engl_27_8


Green tissues of the pericarps and coats at the middle maturation stage transmitted blue and red light (see Supplementary Material, d, e). However, together, they delayed it; as a result, the cotyledons received mainly light in the range of 500–650 and 700–770 nm and a small amount of light in the range of 600–700 nm (see Supplementary Material, e). The high light transmittance (“transparency”) of the pericarp is illustrated in Fig. 1, a.

**Fig. 2.**
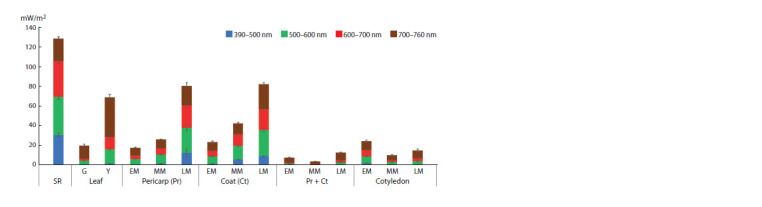
Light transmittance in the tissues of P. sativum at the early (EM), middle (MM), and late (LM) maturation stages. Blue, green, red, and brown bars show the spectral radiance density (mW/m2) in the ranges of 390–500, 500–600, 600–700, and 700– 760 nm, respectively. SR – solar radiation. Data are presented as the means ± standard deviation obtained in three biological replicates.

**Fig. 3.**
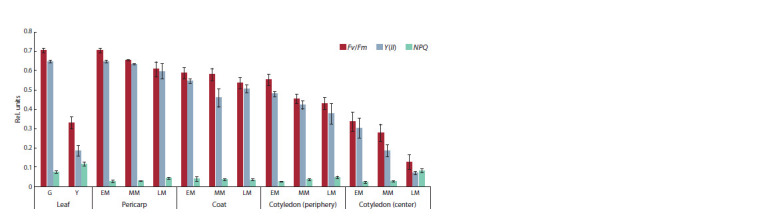
Photochemical activity in the tissues of P. sativum at the early (EM), middle (MM), and late (LM) maturation stages. Fv/Fm and Y(II ) are the maximum and effective quantum yields of the PSII photochemical efficiency, respectively. NPQ is the non-photochemical quenching of chlorophyll fluorescence. Data are presented as the mean ± standard deviation obtained in three biological replicates. A two-way analysis of variance (ANOVA) with replications showed significant changes in the principal factor “photochemical activity” (F(2) = 1282, p < 0.001), the principal factor “plant tissues” (F(13) = 63, p < 0.001), and the interaction between the two factors (F(26.84) = 19, p < 0.001).

We then assessed the dynamics of light transmittance (Fig. 2) and the associated photochemical activity (Fig. 3) during pea seed maturation at the early and middle stages and the beginning of the late stage. The spectral radiance density (SRD) of solar radiation reaching the surface of the leaves and the pericarp averaged 136 mW/m2 (see Fig. 2). The proportions of blue and red light were 32 and 39 mW/m2 (1:1), respectively. We took these values as 100 % and then calculated the percent of the “transmitted” light

The photochemical activity in green leaves was high (Fv/ Fm = 0.71 ± 0.01, and Y(II ) = 0.65 ± 0.01) (see Fig. 3). A green leaf transmitted about 20 mW/m2 (15 %) (see Fig. 2). No blue light was transmitted, and the transmitted red light intensity was about 2 mW/m2 (5 %). As the leaf senesced, chlorophylls degraded, and photochemical activity decreased (Fv/ Fm = 0.33 ± 0.03, and Y(II ) = 0.18 ± 0.03). As a result, 69 mW/m2 (51 %) passed through the senesced yellow leaf, with the proportion of blue and red light increasing to 1.7 mW/m2 (5 %) and 12.7 mW/m2 (32 %), respectively. Leaf senescence was accompanied by an increase in the NPQ (nonphotochemical fluorescence quenching) from 0.07 ± 0.01 up to 0.12 ± 0.01.

Pericarp. At the early stage of seed maturation, the pericarp transmitted 18 mW/m2 (13 %), which is close to the values manifested by the green photosynthetic leaf (see Fig. 2). However, the proportions of blue and red light transmitted by the pericarp were higher, amounting to 0.5 mW/m2 (1.5 %) and 3.5 mW/m2 (8.9 %), respectively. The photochemical activity was increased (equivalent to that of the green leaf). It amounted to 0.69 ± 0.02 for Fv/Fm and 0.68 ± 0.01 for Y(II ) (see Fig. 3). At the middle stage of seed maturation, the amount of light transmitted by the pericarp increased to 26 mW/ m2 (19 %), with the blue and red light reaching 1.8 mW/m2 (5.6 %) and 6.4 mW/m2 (16.4 %), respectively. Meanwhile, the photochemical activity declined slightly (Fv/Fm = 0.65 ± 0.01; Y(II ) = 0.64 ± 0.01). A more significant decrease in Fv/ Fm occurred during the transition to late maturation (Fv/ Fm = = 0.61 ± 0.04; Y(II ) = 0.60 ± 0.04). At the same time, the pericarp tissue turned even more translucent: the transmitted light increased to 81 mW/m2 (60 %), with 12.6 mW/m2 (39 %) for blue light and 23.1 mW/m2 (59 %) for red light.

In the seed coat, Fv/Fm and Y(II ) did not change significantly from the early to late stage of maturation but were slightly lower than in the pericarp (see Fig. 3). The total transmitted light amount increased from 24 to 83 mW/m2 (18 to 61 %), the amount of blue light, from 1.5 to 9.4 mW/m2 (4.6 to 24.1 %), and red light, from 5.7 to 21.4 mW/m2 (17.8 to 54.8 %) (see Fig. 2).

Cotyledons. “Pericarp + coat” (P + C) characterizes the amount and spectral composition of the light transmitted through the pericarp and coat and reaching the cotyledons (see Fig. 2). At the photochemically active early and middle stages of seed maturation, the amount of transmitted light never exceeded 8 mW/m2 (6 %), with no blue light, and less than 1 mW/m2 (less than 2 %) of red light (see Fig. 2). Surprisingly, photochemical processes took place in the cotyledons even at such low levels of light energy, albeit with low efficiency. The photochemical activity of the cotyledons was assessed externally (at the periphery) and internally (by longitudinal sectioning). At early maturation, Fv/Fm was 0.55 ± 0.03 at the periphery of the cotyledons and 0.33 ± 0.05 inside them (see Fig. 3). At late maturation, Fv/Fm decreased to 0.43 ± 0.03 at the periphery of the cotyledons and 0.13 ± 0.04 in their center. Y(II ) showed similar dynamics but was lower than Fv/Fm. At this stage, we also observed an increase in the NPQ index, characterizing the non-photochemical fluorescence quenching (from 0.02 ± 0.01 to 0.08 ± 0.01).

It was interesting to note that the cotyledons were also transparent to sunlight. At early maturation, they transmitted 25 mW/m2 (18 %), which was about the same as for the pericarp and coat (see Fig. 2). At the same time, they transmitted more blue light (2.2 mW/m2, 6.9 %) and red light (6.4 mW/ m2, 16.4 %). Later, however, as reserve nutrients accumulated, the level of transmitted light decreased to 10–15 mW/m2 (8–10 %).

## Discussion

Seeds produce a wide variety of storage compounds that directly (as food) or indirectly (as animal feed) provide up to 70 % of the calories required by humans (Sreenivasulu, Wobus, 2013; Ingram et al., 2018; Mattana et al., 2022). The synthesis of storage compounds, limited by the low oxygen diffusion through seed tissues, is complex without significant energy and assimilates provided by photosynthesis (Walter, Kromdijk, 2021). Furthermore, seed development in many plant species (the so-called chloroembryophytes) requires not only the photosynthesis in the leaves of the mother plant but also photochemical processes of ATP and NADPH+ synthesis in the embryos (Borisjuk et al., 2005; Weber et al., 2005; Puthur et al., 2013; Smolikova, Medvedev, 2016; Smolikova et al., 2018, 2020; Sela et al., 2020; Shackira et al., 2022; Cho et al., 2023).

We have previously shown that in the P. sativum embryos, the synthesis of chlorophylls and the appearance of chloroplasts with a well-developed granum structure occur at the earliest stages of embryogenesis (Smolikova et al., 2018, 2020). In other words, even though developing pea seeds have covering tissues (pericarps and coats) shielding them from sunlight, they receive sufficient light for synthesizing chlorophylls and transforming plastids into chloroplasts. However, the question remained about the spectral range of light that reaches green embryos and the intensity at which their photochemical activity occurs.

In this study, we examined pea seeds at the early, middle, and late stages of maturation (see Fig. 1). We carried out a comparative analysis of light transmission (see Fig. 2) and photochemical activity (see Fig. 3) in leaves, pericarps, coats, and cotyledons of developing seeds using the spectroradiometer and the PAM fluorometer

The 400–700 nm range is known to be the one in which green leaves absorb about 85 %, reflect about 10 %, and transmit about 5 % of light (Atwell et al., 1999). However, these values vary considerably depending on the plant species and growing conditions. In our experiments, photochemically active green pea leaves transmitted an average of 15 % of solar radiation (in the 390–760 nm) with no blue light and no more than 5 % red light

The photochemically active pericarp tissue at the early and middle stages of pea seed maturation allowed 13 to 19 % of solar radiation to pass to the seed coat; in this case, the share of blue light ranged from 1.5 to 6 %, and that of red light, from 9 to 16 %. The periphery of developing cotyledons received light in 500–650 nm and 700–770 nm (6 % of solar radiation); blue light was utterly absent, and the amount of red light (620–700 nm) was about 2 %. With the senescence of covering tissues at the late stage of seed maturation, chlorophylls decomposed, and the transmitted red light amount that reached the cotyledons increased.

Interestingly, low light energy failed to stop photochemical processes from occurring even in the center of the cotyledons, although their efficiency was low. The photochemical activity of cotyledons was recorded in the almost complete absence of blue light, at a low level of red light, and a relatively high level of yellow and green light. At the early stage of seed maturation, the Fv/Fm index was 0.55 ± 0.03 at the periphery of the cotyledons and 0.33 ± 0.05 inside them (see Fig. 3).

How can we explain the photochemical activity in cotyledons at low light radiance densities and spectral ranges untypical for leaf photosynthesis? We hypothesize that green light may partially compensate for the lack of blue light in the cotyledons of developing seeds and thus increase the amount of light energy. Such compensation is likely to occur in the range of 500–550 nm, and the carotenoids present in embryos can absorb this light energy (Smolikova, Medvedev, 2015).

Light in the range of 500–600 nm was believed to be of minor importance in plant biology for a long time. Indeed, plant leaves do not absorb photons uniformly across the entire range of photosynthetically active radiation (PAR), and the spectral absorption of green light by chloroplast photosystems is much lower than in the case of blue and red light (Kume, 2017). However, evidence has emerged in recent years that green light is not only absorbed by plant tissues but is involved in the regulation of many physiological reactions (Golovatskaya, Karnachuk, 2015; Smith et al., 2017). Some authors assumed that the blue and red spectra are predominantly absorbed by the surface cells of the leaf’s columnar mesophyll, while green light can penetrate deeper layers of the leaf tissue, promoting the excitation of photosystems in spongy mesophyll cells (Nishio, 2000; Terashima et al., 2009; Brodersen, Vogelmann, 2010).

J. Liu and M.W. van Iersel (Liu, van Iersel, 2021) assessed the quantum yield of assimilated CO2 (QY) in lettuce leaves grown under different spectral ranges of illumination (blue, green, and red) and at different photosynthetic photon radiance densities (PPFD) (30–1300 mmol photons/m2/s). It turned out that the QY was higher at a high PPFD under green light illumination than under blue or red-light illumination. The authors speculated that it was because, under intense illumination, green light is more evenly distributed within the leaf. Experiments with sunflower leaves also showed that adding green light under moderate to intense white-light illumination is more effective in stimulating photosynthesis than red light addition (Terashima et al., 2009).

A recently published study (Lv et al., 2022) on Zingiber officinale Roscoe plants demonstrated that the addition of green light to the white spectrum not only induced an increase in the Fv/Fm and Y(II ) photochemical parameters but also led to an increase in the number of starch grains and leaf thickness. Intense green light, on the one hand, led to an increased rate of electron flow along the electron transport chain of PS II and, on the other hand, failed to trigger the accumulation of reactive oxygen species (ROS), usually occurring under light stress caused by red light. The authors attributed this phenomenon to more efficient thermal dissipation of excess green light energy.

Our experiments established that PAR reaches the periphery of developing cotyledons, which includes the green part of the spectrum and a small amount of red light. It is possible that green light penetrating through covering tissues to the embryo can affect carbon assimilation efficiency and serve as a good argument in favor of using green wavelengths in crop cultivation. However, this hypothesis requires additional research.

## Conclusion

The data obtained make it possible to better understand the mechanisms of photochemical processes in seed embryos under low light intensity. We believe that the intensity of embryonic photochemical reactions significantly affects the efficiency of reserve nutrient accumulation and, therefore, can be considered a marker for plant breeders seeking to produce seeds with improved nutritional qualities. Research efforts to optimize the production of high-quality seeds by enhancing photochemical activity in their embryos through varying light parameters are also promising.

## Conflict of interest

The authors declare no conflict of interest.
